# *TERT* promoter mutation associated with multifocal phenotype and poor prognosis in patients with *IDH* wild-type glioblastoma

**DOI:** 10.1093/noajnl/vdaa114

**Published:** 2020-09-01

**Authors:** Zensho Kikuchi, Ichiyo Shibahara, Tetsu Yamaki, Ema Yoshioka, Tomoko Shofuda, Rintaro Ohe, Ken-ichiro Matsuda, Ryuta Saito, Masayuki Kanamori, Yonehiro Kanemura, Toshihiro Kumabe, Teiji Tominaga, Yukihiko Sonoda

**Affiliations:** 1 Department of Neurosurgery, Faculty of Medicine, Yamagata University, Yamagata City, Yamagata, Japan; 2 Department of Neurosurgery, Kitasato University School of Medicine, Sagamihara City, Kanagawa, Japan; 3 Department of Biomedical Research and Innovation, Institute for Clinical Research, National Hospital Organization Osaka National Hospital, Osaka, Japan; 4 Department of Pathological Diagnostics, Faculty of Medicine, Yamagata University, Yamagata City, Yamagata, Japan; 5 Department of Neurosurgery, Tohoku University Graduate School of Medicine, Sendai City, Miyagi, Japan

**Keywords:** distant, glioblastoma, *IDH* wild type, multifocal, *TERT* promoter

## Abstract

**Background:**

Although mutations in the promoter region of the telomerase reverse transcriptase (*TERT*p) gene are the most common alterations in glioblastoma (GBM), their clinical significance remains unclear. Therefore, we investigated the impact of *TERT*p status on patient outcome and clinicopathological features in patients with GBM over a long period of follow-up.

**Methods:**

We retrospectively analyzed 153 cases of GBM. Six patients with isocitrate dehydrogenase 1 (*IDH1*) or *H3F3A* gene mutations were excluded from this study. Among the 147 cases of *IDH* wild-type GBM, 92 (62.6%) had the *TERT*p mutation. Clinical, immunohistochemical, and genetic factors (*BRAF*, *TP53* gene mutation, CD133, ATRX expression, *O*^6^-methylguanine-DNA methyltransferase [*MGMT*] promoter methylation) and copy number alterations (CNAs) were investigated.

**Results:**

GBM patients with the *TERT*p mutation were older at first diagnosis versus those with *TERT*p wild type (66.0 vs. 60.0 years, respectively, *P* = .034), and had shorter progression-free survival (7 vs. 10 months, respectively, *P* = .015) and overall survival (16 vs. 24 months, respectively, *P* = .017). Notably, magnetic resonance imaging performed showed that *TERT*p-mutant GBM was strongly associated with multifocal/distant lesions (*P* = .004). According to the CNA analysis, *TERT*p mutations were positively correlated with *EGFR* amp/gain, *CDKN2A* deletion, and *PTEN* deletion; however, these mutations were negatively correlated with *PDGFR* amp/gain, *CDK4* gain, and *TP53* deletion.

**Conclusions:**

*TERT*p mutations were strongly correlated with multifocal/distant lesions and poor prognosis in patients with *IDH* wild-type GBM. Less aggressive GBM with *TERT*p wild type may be a distinct clinical and molecular subtype of *IDH* wild-type GBM.

**Key Points**

- *TERT*p mutations strongly correlated with multifocal/distant lesions and poor prognosis in patients with *IDH* wild-type GBM.
- The *IDH* wild-type GBM with and without *TERT*p mutations may be a distinct clinical and molecular subtype.

**Importance of the Study**

Mutations in the promoter region of the telomerase reverse transcriptase (*TERT*p) gene are the most common mutations in isocitrate dehydrogenase (*IDH*) wild-type glioblastoma (GBM). While *TERT*p mutations are correlated with poor prognosis, aggressive clinicopathological characteristics, and metastasis in other cancers, their clinical significance in GBM remains unclear. Here, we analyzed GBMs to determine whether the *TERT*p status is associated with other clinical and molecular factors. Particularly, this study focused on whether multifocal/distant lesions were observed during the clinical course. In this study, we demonstrated that *TERT*p-mutant GBMs are strongly associated with the prognosis and multifocal/distant lesions during a long follow-up period. In addition, *TERT*p mutation was positively correlated with *EGFR* amp/gain, *CDKN2A* deletion, and *PTEN* deletion; however, it negatively correlated with *PDGFR* amp/gain, *CDK4* gain, and *TP53* deletion. Less aggressive GBM with *TERT*p wild type could be distinct clinical and molecular subtype of *IDH* wild-type GBM.

Glioblastoma (GBM) is the most common primary malignant tumor affecting the central nervous system in adults.^1^ Despite of radical surgery combined with concomitant chemoradiation therapy based on temozolomide, the median survival of patients is approximately 18 months.^2^

According to the World Health Organization revised neuropathological criteria, these tumors are divided into 2 categories, namely isocitrate dehydrogenase (*IDH*) wild-type and *IDH*-mutant GBMs. In addition, recent reports indicated that 70%–80% of GBM genomes harbor either C228T or C250T mutations in the promoter region of the telomerase reverse transcriptase (*TERT*p) gene.^3,4^ These mutations are associated with enhanced telomere maintenance.^5–7^ Although several studies reported the prognostic significance of *TERT*p mutation in patients with GBM, its clinical and pathological roles remain unclear.^3–6^

Recently, GBM patients with unmethylated *O*^6^-methylguanine-DNA methyltransferase (*MGMT*) and *TERT*p mutation have a worse prognosis than those with *TERT*p wild type.^3,8^ However, the mechanism of interaction of *TERT*p mutation and *MGMT* promoter methylation is not well established.

Regarding imaging analysis, necrosis detected through magnetic resonance imaging (MRI) has been reported to indicate the presence of *TERT*p mutation.^9^ However, predicting the *TERT*p status by preoperative imaging study alone remains difficult.

A recent systematic review and meta-analyses stated that the incidence of solitary GBM is 83%.^10^ Other previous studies showed that 20% of patients with GBM had multiple lesions and their prognosis was worse than that recorded in patients with a single lesion.^11^

In this study, we analyzed GBMs to determine whether the *TERT*p status was associated with other clinical and molecular factors. Particularly, this study utilized MRI to determine the development of multifocal/distant lesions during the clinical course.

## Materials and Methods

### Patients and Samples

This retrospective study was conducted with the approval of the Ethics Committees of the Tohoku University School of Medicine and Yamagata University School of Medicine. Written informed consent was provided by all patients prior to their participation in the study.

**Figure 1.**
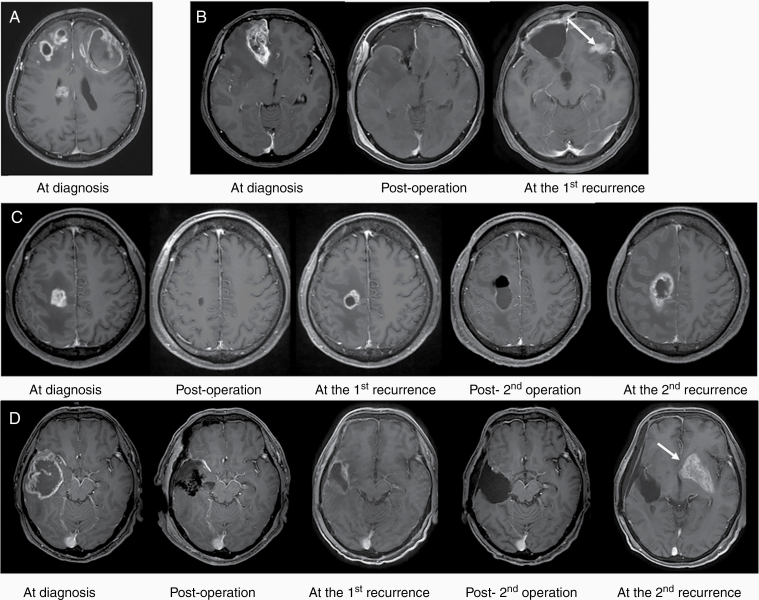
Definition of multifocal lesion. Representative gadolinium-enhanced MRI scans of patients treated at Yamagata University Hospital. The scans were obtained at diagnosis and after surgery, and at the first and second recurrence. (A) Multifocal lesions at diagnosis. (B) Multifocal/distant lesion at first recurrence. Eight months after surgery, an enhanced lesion was observed at a location distant from the initial lesion (arrow). (C) Local recurrence during the entire follow-up period. (D) Multifocal/distant lesion at second recurrence. Seven months after surgery, local recurrence was observed adjacent to the resection cavity. Ten months later, an enhanced lesion was detected at a distant location (arrow).

Between January 2009 and October 2019, a total of 153 patients (89 treated at Yamagata University Hospital [Yamagata cohort] and 64 treated at Tohoku University Hospital [Tohoku cohort]) were analyzed. All patients met the following inclusion criteria: (1) diagnosis of GBM, World Health Organization grade IV; (2) no history of lower-grade tumors; (3) availability of genomic DNA; and (4) availability of information regarding events, such as recurrence or death during the follow-up period, or absence of such events for ≥12 months of follow-up. Patients who had previously undergone biopsies were excluded from the study. Tumor specimens were obtained from a lesion that exhibited enhancement on gadolinium-enhanced MRI and immediately stored at −80°C until DNA extraction (Figure 1).

### Classification of GBM According to Preoperative MRI

MRI sequences were acquired on a 1.5-T or 3.0-T scanner and typically included axial T1-weighted, T2-weighted fast spin-echo, and fluid-attenuated inversion-recovery sequences as well as a postcontrast 3-dimensional spoiled gradient-recalled acquisition in the steady state T1-weighted sequence. Contrast-enhanced lesions (CELs) were assessed to clarify whether they were in contact with the subventricular zone, as previously described.^12^

### Definition of Multifocal/Distant Lesions

One or more enhancing noncontiguous lesions >1 cm distant from the original tumor on preoperative MRI were defined as multifocal/distant lesions at diagnosis.^13^

In addition, as previously reported, “multifocal/distant lesions at recurrence” were defined as distant or multifocal recurrence. Recurrence was characterized by the development of new CEL centered >3 cm distant from the primary resection cavity or at the margins of the primary residual tumor, or at more than 1 site, with each lesion having a well-defined border and the patient exhibiting normal brain signals.^14,15^

### Clinical Parameters

The clinical profiles of patients were obtained from their medical records. The majority of patients underwent radical surgery followed by chemotherapy (nimustine hydrochloride [ACNU] or temozolomide) and radiotherapy. Total surgical resection was defined as the disappearance of CEL according to pre- and postoperative gadolinium-enhanced MRI studies. In cases in which the primary tumor recurred, patients underwent salvage surgery, second-line chemotherapy, radiotherapy, or palliative therapy. The Ki-67 labeling index was determined by immunohistochemical staining of resected specimens with the Ki-67 antigen (Dako, Agilent Technologies). We also analyzed the expression of CD133 (Miltenyi Biotec), p53 (Dako, Agilent Technologies), and ATRX (Abcam) by immunohistochemical staining. The expression of CD133 in 144 patients among the Yamagata and Tohoku cohorts was previously reported.^16,17^

### Prognosis

Progression-free survival (PFS) was defined as the interval between the day of first surgery and the day of recurrence detection on MRI scans. Overall survival (OS) was defined as the time between the day of the first operation and the day of death or final follow-up.

### Molecular Analysis

Genomic DNA was extracted with the QIAamp DNA mini kit (Qiagen), according to the instructions provided by the manufacturer. The isocitrate dehydrogenase1/2 (*IDH1/2*), *H3F3A*, *HIST1H3B*, *TP53*, *BRAF*, and *TERT*p genes were amplified via polymerase chain reaction (PCR), and sequencing was conducted as previously described.^18,19^ In the *MGMT* promoter methylation analysis, we performed methylation-specific PCR or quantitative methylation-specific PCR following the bisulfite modification of tumor DNA.^19^ To assess copy number alterations (CNAs), we performed Multiplex Ligation-dependent Probe Amplification (MLPA) using the SALSA MLPA KIT P105 (version D2), in accordance with the manufacturer’s protocol (MRC Holland).^20^ The P105 kit is designed to detect CNAs typically found in gliomas and includes probes against the *PDGFRA*, *EGFR*, *CDKN2A*, *PTEN*, *TP53*, *CDK4*, *MDM2*, and *NFKBIA* genes. Based on the previous publications, the CNA categories were classified according to the following thresholds: homozygous deletion (*x* ≤ 0.4), hemizygous deletion (0.4 < *x* ≤ 0.7), gain (1.3 ≤ *x* < 2.0), and amplification (*x* ≥ 2.0).^20,21^ We used OncoPrinter, a tool provided by the cBioPortal for Cancer Genomics (cbioportal.org/oncoprinter), to visualize and analyze our data with some modifications.^22,23^

### Statistical Analysis

Statistical analyses were performed using the SPSS (IBM Japan) software. The relationship between 2 variables was evaluated using the Mann–Whitney *U* test and Fisher’s exact test. Estimates of PFS and OS were calculated with the Kaplan–Meier method, and the Log-rank (Mantel–Cox) test was used to evaluate differences between the groups. Cox regression was used for the multivariate analysis. The significance level was set at *P* < .05.

## Results

### Population and Tumor Characteristics on MRI

**Figure 2.**
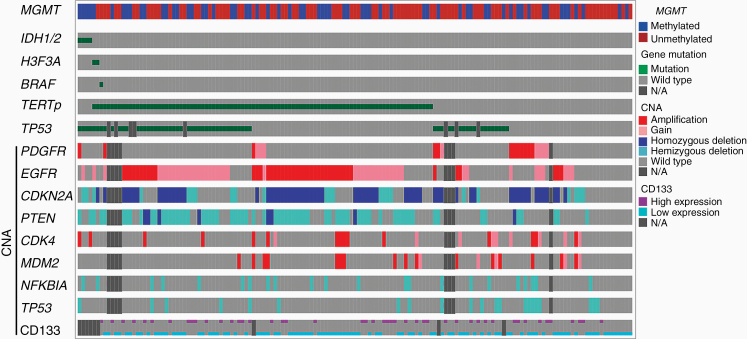
Genetic distribution in 153 GBMs. Mutations, CNAs, and methylation were generated and visualized by OncoPrinter via the cBioPortal for Cancer Genomics (cbioportal.org/oncoprinter) with some modifications.^22,23^ The diagram shows the landscape of the molecular characteristics of GBMs, which are sorted by *IDH*, *H3F3A*, and *TERT*p mutations. N/A, not available.

A total of 153 patients, including 82 males and 71 females with a median age of 63 years (range: 27–86 years) and median preoperative Karnofsky Performance Status of 80 (range: 30–100), were included in the present study. Patients in the Yamagata cohort were older than those in the Tohoku cohort (*P* < .001) (Supplementary Table 1). Genomic DNA and paraffin-embedded samples were obtained from all patients. The median duration of the follow-up period was 17 months (range: 1–152 months), and 119 patients (77.8%) expired. Total surgical resection was achieved in 96 patients (62.7%). In this group, *IDH1*, *H3F3A*, and *BRAF* gene mutations were detected in 4 (2.6%), 2 (1.3%), and 1 patient (0.65%), respectively; however, neither *IDH2* nor *HIST1H3B* gene mutations were detected. *TERT*p gene mutations were detected in 92 patients (60.1%), including 65 (42.5%) and 27 (17.6%) with C228T and C250T mutations, respectively. Although the frequency of *TERT*p gene mutations in the Yamagata cohort was higher than that in the Tohoku cohort (*P* = .019, Supplementary Table 1), there was no significant difference in the mutation frequency in older patients (age ≥60) between the 2 cohorts (*P* = .348) (data not shown). *MGMT* gene promoter methylation was found in 62 patients (40.5%). Postoperative treatments consisted of radiation alone for 6 patients, while the remaining 147 patients received combined radiation and chemotherapy with temozolomide (*n* = 123), ACNU (*n* = 14), or other agents (*n* = 10). Bevacizumab was administered as first- and second-line therapy in 1 and 50 patients, respectively. There were no significant differences observed in PFS and OS between patients treated with ACNU and temozolomide (data not shown). *TP53* gene mutations and/or strong immunoreactivity of p53 were found in 63 patients (43.4%) (Figure 2). Eleven of 88 patients (12.5%) displayed the loss of ATRX expression. The major CNAs frequently observed in 139 GBMs included *EGFR* amp/gain (66.2%), *CDKN2A* deletion (60.4%), and *PTEN* deletion (51.8%) (Figure 2 and Table 1).

**Table 1.** Relationships Between *TERT*p Status and Other Prognostic Factors

			Total (*n* = 147)	*TERT*p wild type (*n* = 55)	*TERT*p mutant (*n* = 92)	*P*
Sex, female, *n* (%)			66 (44.8)	27 (49.1)	39 (42.4)	.494^b^
Age, y, median (range)			64 (27–86)	60 (27–82)	66 (32–86)	**.034** ^**a**^
Preoperative KPS ≥80, *n* (%)			84 (60.0)	27 (51.9)	57 (64.8)	.155^b^
Gross total resection, *n* (%)			93 (63.2)	33 (60.0)	60 (65.2)	.597^b^
CD133 expression, mean (%)			12.7 ± 12.9	12.1 ± 11.1	12.9 ± 13.8	.729^a^
Ki-67 labeling index, mean (%)			33.8 ± 17.9	34.8 ± 17.9	33.2 ± 18.0	.477^a^
Multifocal/distant lesions						
At diagnosis, *n* (%)			21 (14.3)	4 (7.3)	17 (18.5)	.087^b^
At recurrence, *n* (%)			45 (30.6)	12 (21.8)	33 (35.9)	.096^b^
At the first recurrence, *n* (%)			30 (20.4)	8 (14.5)	22 (23.9)	.208^b^
At the second recurrence, *n* (%)			15 (10.2)	4 (7.3)	11 (12.0)	.415^b^
Total, *n* (%)			66 (44.9)	16 (29.1)	50 (54.3)	**.004** ^**b**^
*MGMT* gene promoter methylation, *n* (%)			57 (38.8)	19 (34.5)	38 (41.3)	.485^b^
*TP53* gene mutation, *n* (%)			57 (40.7)	19 (37.3)	38 (42.7)	.477^b^
Loss of ATRX expression, *n* (%)			11 (12.5)	6 (22.2)	5 (8.2)	.085^b^
CNA	*PDGFR*	Amp, *n* (%)	11 (7.9)	9 (17.6)	2 (2.3)	**.002** ^**b**^
		Gain, *n* (%)	8 (5.8)	5 (9.8)	3 (3.4)	.143^b^
		Amp/gain, *n* (%)	19 (13.7)	14 (27.5)	5 (5.7)	**.001** ^**b**^
	*EGFR*	Amp, *n* (%)	46 (33.1)	8 (15.7)	38 (43.2)	**.001** ^**b**^
		Gain, *n* (%)	46 (33.1)	7 (13.7)	39 (44.3)	**<.0001** ^**b**^
		Amp/gain, *n* (%)	92 (66.2)	15 (29.4)	77 (87.5)	**<.0001** ^**b**^
	*CDKN2A*	Homo, *n* (%)	61 (43.9)	20 (39.2)	41 (46.6)	.479^b^
		Hemi, *n* (%)	23 (16.5)	5 (9.8)	18 (20.5)	.154^b^
		Deletion, *n* (%)	84 (60.4)	25 (49.0)	59 (67.0)	**.048** ^**b**^
	*PTEN*	Homo, *n* (%)	8 (5.8)	1 (2.0)	7 (8.0)	.258^b^
		Hemi, *n* (%)	64 (46.0)	8 (15.7)	56 (63.6)	**<.0001** ^**b**^
		Deletion, *n* (%)	72 (51.8)	9 (17.6)	63 (71.6)	**<.0001** ^**b**^
	*CDK4*	Amp, *n* (%)	13 (9.4)	4 (7.8)	9 (10.2)	.768^b^
		Gain, *n* (%)	9 (6.5)	7 (13.7)	2 (2.3)	**.012** ^**b**^
		Amp/gain, *n* (%)	22 (15.8)	11 (21.6)	11 (12.5)	.227^b^
	*MDM2*	Amp, *n* (%)	16 (11.5)	6 (11.8)	10 (11.4)	1.000^b^
		Gain, *n* (%)	5 (3.6)	4 (7.8)	1 (1.1)	.061^b^
		Amp/gain, *n* (%)	21 (15.1)	10 (19.6)	11 (12.5)	.327^b^
	*NFKBIA*	Hemi, *n* (%)	20 (14.4)	8 (15.7)	12 (13.6)	.804^b^
	*TP53*	Hemi, *n* (%)	23 (16.5)	16 (31.4)	7 (8.1)	**.001** ^**b**^
		Mut/Hemi, *n* (%)	12 (8.6)	7 (13.7)	5 (5.7)	.124^b^
SVZ-positive, *n* (%)			66 (44.9)	25 (45.5)	41 (44.6)	1.000^b^

Amp, amplification; Hemi, hemizygous deletion; Homo, homozygous deletion; KPS, Karnofsky Performance Status; Mut, mutation; SVZ, subventricular zone. *P* values <0.05 are in bold.

^a^Mann–Whitney test.

^b^Fisher’s exact test.

### Correlation Analyses Between the *TERT*p Status and Other Prognostic Factors

Six patients with *IDH1* or *H3F3A* mutations were excluded from this study. Therefore, we analyzed 147 GBM patients with *IDH* wild type to determine the factors correlated with the *TERT*p mutation. The median age was higher in GBM patients with *TERT*p mutation than those with *TERT*p wild type (*P* = .034) (Table 1).

In terms of MRI characteristics, 21 of the 147 patients (14.3%) had multifocal/distant lesions at diagnosis (Table 1). During the follow-up, 129 patients (87.7%) experienced the first recurrence, which included local recurrence and multifocal/distant recurrence in 99 (67.3%) and 30 (20.4%) patients, respectively. Among the patients with a well-controlled first recurrent lesion, 15 patients (10.2%) had new multifocal/distant lesions at second recurrence. Neither local nor distal recurrence was observed at the time of the last observation in the remaining 18 patients (12.3%).

Although multifocal/distant lesions at diagnosis or recurrence were weakly correlated with *TERT*p mutations (*P* = .087 and *P* = .096, respectively), these lesions were significantly more common in patients with *TERT*p-mutant GBM than in patients with *TERT*p wild-type GBM during the entire follow-up period (*P* = .004, Table 1).

The loss of ATRX expression occurred more frequently in *TERT*p wild-type GBM; however, this difference was not significant (*P* = .085, Table 1). *EGFR* amp/gain, *CDKN2A* deletion, and *PTEN* deletion were significantly associated with *TERT*p mutations (*P* < .0001, *P* = .048, and *P* < .0001, respectively, Figure 2 and Table 1). Conversely, *PDGFR* amp/gain, *CDK4* gain, and *TP53* hemizygous deletion were more frequently observed in *TERT*p wild-type GBM (*P* = .001, *P* = .012, and *P* = .001, respectively, Figure 2 and Table 1).

### Univariate Analysis for the Prediction of PFS and OS

**Figure 3.**
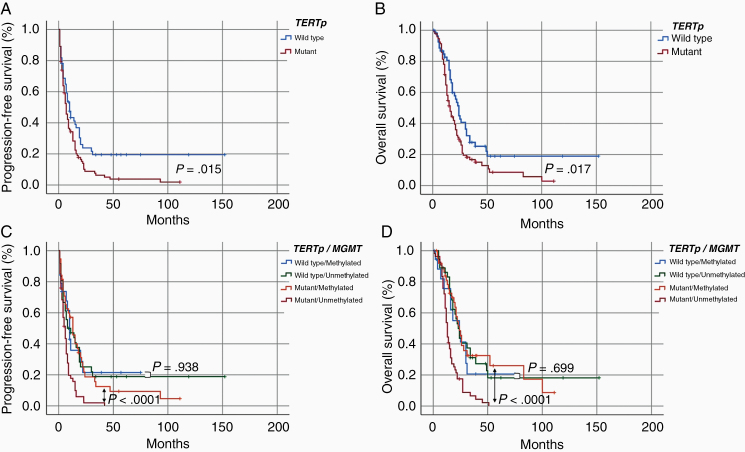
(A and B) Kaplan–Meier curves based on the *TERT*p mutation in patients with *IDH* wild-type GBM. (A) PFS. (B) OS. (C and D) Kaplan–Meier curves based on the combination of *TERT*p mutation and *MGMT* promoter methylation in patients with *IDH* wild-type GBM. (C) PFS. (D) OS.

The median PFS and OS for the patients with *IDH* wild-type GBM were 8 and 18 months, respectively (Table 2). Based on the Kaplan–Meier analysis, longer PFS and OS were correlated with *TERT*p wild type (*P* = .015 and *P* = .017, respectively) (Figure 3A and B; Table 2), gross total resection (*P* < .001 and *P* <.001, respectively) (Table 2), *MGMT* gene promoter methylation (*P* = .037 and *P* = .015, respectively) (Table 2), *CDK4* amp/gain (*P* = .015 and *P* = .042, respectively), and local lesions (*P* = .006 and *P* = .001, respectively) (Table 2). The female sex was associated with longer PFS (*P* = .047) (Table 2).

**Table 2.** Clinical and Genetic Parameters Affecting PFS and OS in Primary GBM

Parameters	No. of patients (*n* = 147)	PFS		OS	
		Median (months)	P*	Median (months)	P*
	147	8		18	
*TERT*p status					
Mutated	92	7		16	
Wild type	55	10	**.015**	24	**.017**
Sex					
Female	66	9		22	
Male	81	7	**.047**	16	.055
Age at diagnosis					
<60 years	52	8		18	
*>*60 years	95	7	.172	19	.115
Preoperative KPS					
*>*80	84	8		21	
<80	56	7	.725	15	.294
Surgery					
Gross toral resection	93	11		23	
Absence of gross total resection	54	4	**<.001**	11	**<.001**
Ki-67 labeling index					
Low (<30%)	55	8		20	
High (>30%)	68	7	.212	16	.061
CD133 expression					
Low (<15%)	97	8		21	
High (*>*15%)	47	7	.480	17	.146
*MGMT*					
Methylated	57	13		24	
Unmethylated	90	7	**.037**	16	**.015**
*PDGFR*					
Amp/gain	19	10		17	
Retain	120	8	.916	20	.669
*EGFR*					
Amp/gain	92	7		17	
Retain	47	10	.060	24	.142
*CDKN2A*					
Deletion	84	9		17	
Retain	55	8	.522	21	.350
*PTEN*					
Deletion	72	9		19	
Retain	67	8	.281	19	.497
*CDK4*					
Amp/gain	22	19		34	
Retain	117	7	**.015**	18	**.042**
*MDM2*					
Amp/gain	21	10		24	
Retain	118	8	.795	18	.368
*NFKBIA*					
Deletion	20	13		21	
Retain	119	8	.802	18	.662
*TP53*					
Mut/deletion	68	10		19	
Wild type	76	7	.054	19	.580
SVZ					
Positive	66	7		16	
Negative	74	8	.952	21	.267
Numbers of lesion					
Multifocal/distant lesions	66	7		16	
Local lesion	81	9	**.006**	23	**.001**
Cohort site					
Yamagata	88	7		17	
Tohoku	59	9	.137	22	.107

SVZ, subventricular zone. *P* values <0.05 are in bold.

*Log-rank test.

To determine whether the *TERT*p mutation was negatively correlated with PFS and OS in the non-multifocal/distant group, we analyzed the survival of the 81 patients in the non-multifocal/distant group. The median PFS and OS were 9 and 23 months, respectively, with no significant correlation of PFS and OS with the *TERT*p mutation (*P* = .129 and *P* = .148, respectively) (data not shown).

We also investigated the prognostic value of *TERT*p mutation in combination with *MGMT* promoter methylation. Among patients with *TERT*p mutation, unmethylated *MGMT* was significantly associated with poor PFS and OS (*P* < .0001 and *P* < .0001, respectively) (Figure 3C and D). However, among patients with *TERT*p wild type, there was no significant difference of PFS and OS between patients with and without *MGMT* promotor methylation (*P* = .938 and *P* = .699, respectively) (Figure 3C and D).

### Factors Associated With Multifocal/Distant Lesions

We investigated several factors to determine whether they correlated with multifocal/distant lesions. As shown in Supplementary Table 2, *TERT*p mutations, the expression of CD133, and *PTEN* deletion were significantly associated with multifocal lesions (*P* = .004, *P* = .004, and *P* = .004, respectively).

### Multivariate Analysis of Prognostic Factors

The factors included in the multivariate analysis for PFS and OS were *TERT*p status, sex, age, extent of resection, Ki-67 labeling index, *MGMT* gene promoter methylation, *CDK4* amp/gain, number of lesions, and cohort site. We found that *TERT*p mutation, absence of gross total resection, and *MGMT* gene promoter unmethylation were independent unfavorable prognostic factors for PFS (hazard ratio [HR]: 2.0, 95% confidence interval [CI]: 1.2–3.3, *P* = .006; HR: 2.2, 95% CI: 1.3–3.5, *P* = .002; and HR: 2.0, 95% CI: 1.3–3.0, *P* = .002, respectively) (Table 3). *TERT*p mutations (HR: 2.0, 95% CI: 1.2–3.3, *P* = .010), absence of total resection (HR: 2.9, 95% CI: 1.7–4.8, *P* < .001), and *MGMT* gene promoter unmethylation (HR: 2.2, 95% CI: 1.4–3.5, *P* = .001) were independent unfavorable prognostic factors for OS.

**Table 3.** Multivariate Analysis of Independent Prognostic Factors Associated With PFS and OS

Parameters	PFS			OS		
	HR	95% CI	P*	HR	95% CI	*P*
*TERT*p status						
Mutant vs. wild type	2.0	1.2–3.3	**.006**	2.0	1.2–3.3	**.010**
Sex						
Male vs. female	1.3	0.9–2.0	.218	1.4	0.9–2.2	.157
Age						
≥60 vs. <60	1.2	0.8–2.0	.364	1.1	0.7–1.9	.600
Gross total resection						
No vs. Yes	2.2	1.3–3.5	**.002**	2.9	1.7–4.8	**<.001**
Ki-67 labeling index						
≥30 vs. <30	1.4	0.9–2.1	.129	1.5	1.0–2.4	.069
*MGMT*						
Unmethylated vs. methylated	2.0	1.3–3.0	.002	2.2	1.4–3.5	**.001**
*CDK4*						
Amp/gain vs. retain	1.5	0.8–2.8	.261	1.5	0.7–2.9	.284
Number of lesions						
Multifocal/distant vs. local	1.3	0.8–2.0	.327	1.3	0.8–2.2	.241
Cohort site						
Yamagata vs. Tohoku	1.1	0.7–1.7	.656	1.1	0.7–1.8	.589

*P* values <.05 are in bold.

## Discussion

*TERT*p mutation is the most common alteration in GBM; however, the clinical impact of *TERT*p mutations in GBM remains unclear. To understand the poor prognosis of GBM with *TERT*p mutations, we hypothesized that malignant clinical features exist in this group. Long-term follow-up revealed that the cumulative incidence of multiple/distant lesions was significantly higher in GBM with *TERT*p mutations than in patients with *TERT*p wild-type GBM. Conversely, the non-multifocal/distant group did not show any differences in PFS and OS based on *TERT*p status. Therefore, we, for the first time, demonstrated that GBM with *TERT*p mutations has a poor prognosis because of its clinically aggressive behavior. In accordance with this finding, several studies regarding other cancers demonstrated that these mutations were correlated with a poor prognosis, aggressive clinicopathological characteristics, and metastasis.^24–28^ Xing et al. found that *TERT*p mutation strongly correlated with vascular invasion in patients with papillary thyroid cancer.^25^ Yuan et al. reported that thyroid cancer patients with the *TERT*p mutation have a 4-fold higher risk of distant metastasis than those with *TERT*p wild type.^27^

The frequency of *TERT*p mutations in our study was 62.6%, which is lower than that of previous reports from North America and European countries, which reported mutation frequencies of 73%–75% in *IDH* wild-type GBMs.^3–5^ Other reports from Japan also showed relatively low frequencies of *TERT*p mutations among *IDH* wild-type GBM, ranging from 50% to 70%.^6,9,29^ Thus, racial differences in the frequency of *TERT*p mutations may exist. One possible explanation for the low frequency of *TERT*p mutations in the Japanese cohort is that other mechanisms involved with replicative immortality in *TERT*p wild-type GBM. One such mechanism is *TERT*p hypermethylation, and the other is *ATRX* or *SMARCAL1* gene mutation. *TERT*p hypermethylation can aberrantly activate telomerase in cancer,^30^ and the *ATRX* or *SMARCAL1* gene mutations are strongly associated with the maintenance of telomere length, referred to as alternative lengthening of telomeres.^31^ Indeed, our results indicated the frequent loss of ATRX expression in *TERT*p wild-type GBM. The other explanation is potential inclusion of other *IDH* wild-type high grade gliomas such as anaplastic astrocytoma with piloid features.^32^ Although our cases were histologically confirmed as GBM, further molecular testing may be required to classify into novel entities.

The prognostic significance of the *TERT*p mutation remains controversial in patients with GBM.^3,33–35^ In the present study, univariate and multivariate analyses showed that the *TERT*p mutation was significantly associated with both PFS and OS. In accordance with previous reports, we also found that unmethylated GBM with *TERT*p mutations presented a poor prognosis.^3,8^ However, among patients with *TERT*p wild type, there was no significant difference of PFS and OS between patients with and without *MGMT* promotor methylation. The reason may be that GBM tumors with the *TERT*p mutation form multifocal/distant lesions by invading various directions. Nevertheless, those with methylated *MGMT* were sensitive to treatment with alkylating agents, such as temozolomide. Therefore, *TERT*p mutated GBM patients with methylated *MGMT* may survive longer than those with unmethylated *MGMT*.

Recently, GBMs were divided into 2 groups according to the *IDH* mutation status. Although *IDH* mutation is frequently found in lower-grade diffuse glioma, only 5%–10% of patients with GBM had this mutation.^36,37^ In addition, GBM patients with the *IDH* mutation are usually young and diagnosed with progression from a lower grade of diffuse astrocytoma. Thus, *TERT*p mutation, frequently found in GBM is more useful for predicting survival and clinical behavior, such as the pattern of invasion.

Our data showed that *TERT*p mutations were significantly associated with *EGFR* amp/gain, *CDKN2A* deletion, and *PTEN* deletion and were typically found in *IDH* wild-type GBM; conversely, the *TERT*p wild type was associated with *PDGFR* amp/gain, *CDK4* gain, and *TP53* deletion. Recently, Williams et al. reported *TERT*p wild-type GBMs showed frequent *PI3K* pathway and *BAF* complex gene family (*ATRX*, *SMARCA4*, *SMARCB1*, and *ARID1A*) mutations.^38^ Our data also suggest that *TERT*p wild-type GBMs are genetically distinct from *TERT*p-mutant GBMs.

The present study had some limitations. First, since this was a retrospective study, patients were not treated in the same manner. Although we performed a multivariate analysis, differences in treatment may have affected the pattern of recurrence. Second, we demonstrated the malignant features of GBM with the *TERT*p mutation based on clinicopathological characteristics, but patients with oligodendroglioma (the most benign diffuse glioma) also had the *TERT*p mutation.^6^ Third, it has been reported that *PTEN*, *PI3K3A* mutation and the expression of CD133 are associated with distant recurrence in patients with GBM.^16,17,39,40^ In the present study, there was no significant association between CD133 expression and the *TERT*p mutation, but *PTEN* deletion was significantly correlated with *TERT*p mutations and multifocal/distant lesions. The mechanism of invasiveness based on the *TERT*p mutation warrants further investigation.

## Conclusion

We retrospectively investigated whether the *TERT*p mutation was associated with multifocal/distant lesions in GBM. The results suggested that the *TERT*p mutations strongly correlated with the multifocal phenotype and poor prognosis in patients with *IDH* wild-type GBM. We further demonstrated that *TERT*p mutations were significantly associated with *EGFR* amp/gain, *CDKN2A* deletion, and *PTEN* deletion, whereas the *TERT*p wild type was correlated with *PDGFR* amp/gain, *CDK4* gain, and *TP53* deletion. The *IDH* wild-type GBM with and without *TERT*p mutations may be a distinct clinical and molecular subtype.

## Supplementary Material

vdaa114_suppl_Supplementary_Table_1

vdaa114_suppl_Supplementary_Table_2

## Notes

**Authorship Statement**: Experimental design (Z.K., I.S., Y.K., T.T., and Y.S.); implementation (Z.K., I.S., T.Y., E.Y., T.S., Y.K., R.O., K.M., and Y.S.); analysis and interpretation of data (Z.K., I.S., T.Y., E.Y., T.S., R.O., K.M., R.S., M.K., Y.K., T.K., and Y.S.); writing of the manuscript (Z.K., I.S., T.Y., E.Y., T.S., R.O., K.M., R.S., M.K., Y.K., T.K., T.T., and Y.S.).
