# Supplementary material for: TERT promoter mutation associated with multifocal phenotype and poor prognosis in patients with IDH wild-type glioblastoma
Source: Neurooncol Adv. 2020 Sep 1;2(1):vdaa114. doi: 10.1093/noajnl/vdaa114 (PMC7586143; doi:10.1093/noajnl/vdaa114)
Supplement: vdaa114_suppl_Supplementary_Table_1 [file vdaa114_suppl_supplementary_table_1.docx]

|  | Total (n=153) | Yamagata cohort (n=89) | Tohoku cohort (n=64) | *P* |
| --- | --- | --- | --- | --- |
| Sex, female, n (%) | 71 (46.4) | 40 (44.9) | 31 (48.4) | 0.743^b^ |
| Age, y, median (range) | 63 (27-86) | 66 (27-86) | 60 (27-76) | **<0.001^a^** |
| Preoperative KPS ≥80, n (%) | 88 (57.5) | 55 (61.8) | 33 (51.6) | 0.247^b^ |
| Gross total resection, n (%) | 96 (62.7) | 51 (57.3) | 45 (70.3) | 0.127^b^ |
| *IDH1* mutation, n (%) | 4 (2.6) | 1 (1.1) | 3 (4.7) | 0.309^b^ |
| *IDH2* mutation, n (%) | 0 (0) | 0 (0) | 0 (0) | N/A |
| *H3F3A* mutation, n (%) | 2 (1.3) | 0 (0) | 2 (3.1) | 0.173^b^ |
| *HIST1H3B* mutation, n (%) | 0 (0) | 0 (0) | 0 (0) | N/A |
| *BRAF* mutation, n (%) | 1 (0.65) | 1 (1.1) | 0 (0) | 1.000^b^ |
| *TERT*p mutation, n (%) | 92 (60.1) | 61 (68.5) | 31 (48.4) | **0.019^b^** |
| *C228T* mutation, n (%) | 65 (42.5) | 44 (49.4) | 21 (32.8) | **0.047^b^** |
| *C250T* mutation, n (%) | 27 (17.6) | 17 (19.1) | 10 (15.6) | 0.670^b^ |
| *MGMT* gene promoter methylation, n (%) | 62 (40.5) | 38 (42.7) | 24 (37.5) | 0.617^b^ |

Supplementary Table 1.　Population characteristics

Abbreviations: ^a^Mann-Whitney test. ^b^Fisher's exact test. P values <0.05 are in bold, N/A not applicable
