# Supplementary material for: TERT promoter mutation associated with multifocal phenotype and poor prognosis in patients with IDH wild-type glioblastoma
Source: Neurooncol Adv. 2020 Sep 1;2(1):vdaa114. doi: 10.1093/noajnl/vdaa114 (PMC7586143; doi:10.1093/noajnl/vdaa114)
Supplement: vdaa114_suppl_Supplementary_Table_2 [file vdaa114_suppl_supplementary_table_2.docx]

Supplementary Table 2.　Factors associated with multifocal/distant lesions

|  | Multifocal/distant lesions (n=66) | Local lesion (n=81) | *P* |
| --- | --- | --- | --- |
| *TERT*p mutant, n (%) | 50 (75.8) | 42 (51.9) | **0.004^b^** |
| CD133 expression, mean (%) | 15.5+13.8 | 10.3+11.6 | **0.004^a^** |
| *TP53* mut/loss, n (%) | 30 (45.5) | 38 (48.7) | 0.737^b^ |
| *PDGFR* amp/gain, n (%) | 6 (9.4) | 13 (17.3) | 0.219^b^ |
| *EGFR* amp/gain, n (%) | 47 (73.4) | 45 (60.0) | 0.108^b^ |
| *CDKN2A* loss, n (%) | 42 (65.6) | 42 (56.0) | 0.297^b^ |
| *PTEN* loss, n (%) | 42 (65.6) | 30 (40.0) | **0.004^b^** |
| *CDK4* amp/gain, n (%) | 6 (9.4) | 16 (21.3) | 0.064^b^ |
| *MDM2* amp/gain, n (%) | 9 (14.1) | 12 (16.0) | 0.815^b^ |
| *NFKBIA* loss, n (%) | 12 (18.8) | 8 (10.7) | 0.227^b^ |
| Sex, female, n (%) | 27 (40.9) | 39 (48.1) | 0.408^b^ |
| Age, y, median (range) | 65 (32-86) | 63 (27-85) | 0.788^a^ |
| Preoperative KPS ≥80, n (%) | 39 (60.9) | 45 (59.2) | 0.864^b^ |
| Ki-67 labeling index, mean | 34.9+18.5 | 32.9+17.4 | 0.739^a^ |
| *MGMT* gene promoter methylation, n (%) | 31 (47.0) | 26(32.1) | 0.089^b^ |
| SVZ-positive, n (%) | 33 (51.6) | 33 (43.4) | 0.396^b^ |

Abbreviations: ^a^Mann-Whitney test. ^b^Fisher's exact test. *P* values <0.05 are in bold. amp, amplification
